# Computational learning phenotypes are not related to individual differences in resting-state fMRI connectivity

**DOI:** 10.3389/fnins.2026.1720206

**Published:** 2026-05-01

**Authors:** Evan Dastin-van Rijn, Linda Q. Yu, Daniel N. Scott, Yifan Zhao, Ani Eloyan, Alexandre Filipowicz, Joseph W. Kable, Tingyong Feng, Matthew Nassar

**Affiliations:** 1 Department of Biomedical Engineering, University of Minnesota, Minneapolis, MN, United States; 2 Department of Neuroscience, Brown University, Providence, RI, United States; 3 Department of Biostatistics, Brown University, Providence, RI, United States; 4 Department of Psychology, University of Pennsylvania, Philadelphia, PA, United States; 5 Department of Psychology, Southwest University, Chongqing, China; 6 Robert J. and Nancy D. Carney Institute for Brain Science, Brown University, Providence, RI, United States

**Keywords:** computational models, individual differences, latent states, learning, resting state fMRI

## Abstract

People learn from experience, but with considerable individual differences in the degree and type of behavioral adjustments resulting from a given experience. Error driven learning rules provide an elegant framework for explaining both learning behavior and its neural signatures; however, implementing them requires carving the world into so-called “latent states”, that serve as substrates for learning, meaning that the same learning algorithm can produce different sorts of learning given different state representations. Recent theoretical and behavioral work hints that individual differences in learning may reflect differences in how individuals carve their environment into states, with some individuals combining multiple temporal contexts into a single state and others separating these contexts into individuated latent states. Here, we develop a behavioral paradigm and modeling framework to test this idea directly and show in a large cohort of human participants that individuals can be classified into groups according to whether and how they carve temporal contexts into latent states. These behavioral phenotypes impact continual learning, specifically the degree to which individuals avoid interference at context changes or are able to reuse information when encountering a familiar context. We tested whether these behavioral phenotypes related to individual differences in underlying brain connectivity, as measured by resting state-fMRI, but found no evidence for such a relationship. Taken together, this work suggests that learning differences across individuals are attributable to differences in underlying state representations that are not predicted by underlying resting state brain connectivity.

## 1 Introduction

Humans and animals learn dynamically from experience, adjusting behavioral policies in accordance with new observations. Despite the ubiquity of learning, people often differ dramatically in what they learn from the same experience. In some cases, this might be a manner of degree, with some individuals revising beliefs dramatically in response to a new observation and others revising beliefs only minimally (Nassar et al., 2010). In other cases, learning differs qualitatively, for example, in which feature an individual credits for learning (Lamba et al., 2023; Shahar et al., 2021, 2019; Ben-Artzi et al., 2023). The extreme variability in learning behaviors among individuals, along with the critical importance of learning for success over the evolutionary timescale and in every day life, raise questions about both the function and source of this variability.

Normative learning models provide some insight into the possible origins of this variability. Bayesian models view learning as a problem in which past experience is used to infer important underlying latent variables that can serve to predict future experience (Gershman and Niv, 2010; Behrens et al., 2007). Such models define optimal inference in cases where the underlying structure of the environment is known, but also highlight the difficulty of inferring that underlying structure (Piray and Daw, 2021; Wilson et al., 2010).

Although full Bayesian inference on problems of real-world complexity is typically intractable, it can often be approximated by error-driven learning rules, which update predictions trial-to-trial according to prediction errors (Nassar et al., 2010, 2019). One class of error-driven learning rules, reinforcement learning (RL) algorithms, has been shown to closely match human and animal learning as well as its underlying correlates (O'Reilly and Frank, 2006; Schultz et al., 1997; Niv, 2009). RL provides a framework to use a sparse reward prediction error signal to train complex sequential policies, but to do so requires separating situations that might require different actions into different “states”. The same RL algorithm can learn very different things when given different state representations, and thus an important problem in RL is how to carve experience into a useful set of states (Gershman and Niv, 2010). This problem is made more difficult by the fact that human experience is highly contextual and that the outcomes of our actions often depend critically on unobservable aspects of temporal context (FeldmanHall and Nassar, 2021). Recent theoretical work has suggested that the brain might solve this problem using latent state representations that are updated dynamically at transitions in temporal context, preventing interference between contexts and facilitating appropriate reuse of information when returning to a previously experienced context (Yu et al., 2021; Razmi and Nassar, 2022). Here we define a latent state representation as an internal representation of a latent aspect of the environment or task. If the brain were to update such representations at transitions, the dynamics of latent-state representations might be an important source of variability in learning behavior (Zika et al., 2023; Zika, 2023). For example, two individuals might differ in their behavioral response to a given experience because they assigned learning to different context representations. These differences in how individuals assign learning may be reflected in underlying biology. One such possibility is that individuals differ in their resting state brain connectivity, which predisposing them to differences in learning (Reineberg et al., 2015; Keller et al., 2015; Elliott et al., 2019).

Here, we use a predictive inference task with an underlying reversal structure to characterize error-driven learning in a large sample (*N* = 265) of human participants. We build a family of computational models that rely on the same error-driven learning rule but differ in their state representation dynamics and show that individuals can be separated into different groups depending on whether they are best described as (1) learning onto a single state representation, (2) creating a new latent state representation upon observation of surprising outcomes, or (3) reusing a previously created latent state representation where appropriate. Finally, we use resting state fMRI data collected before task performance to test whether categorical differences in latent-state learning are related to resting brain connectivity. We find evidence that individuals differ in their learning strategies and that these differences are captured by computational models that differ in their assumptions about the latent states of the environment. However, we find no evidence that these differences relate to resting state brain connectivity. Taken together, our results suggest that individual differences in learning result from differences in how people carve experience into latent states, and could be interpreted as supporting the notion that these differences reflect cognitive strategies rather than biologically-constrained predispositions.

## 2 Methods

### 2.1 Experimental task

**Figure 1:**
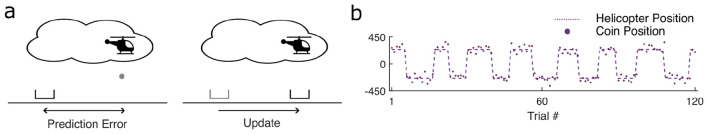
Overview of predictive inference task. **(a)** Stylized representation of the task. On each trial participants observed coins dropped by a helicopter occluded by clouds. Participants had control over a bucket which they used to catch coins dropped by the helicopter. **(b)** Example sequence of helicopter positions and exact locations of corresponding coin drops.

We analyzed behavioral and resting-state fMRI data obtained from 265 participants (ages 17–30, mean 20) who performed a predictive-inference task involving a “helicopter” (McGuire et al., 2014; Kao et al., 2020). In the behavioral task, participants placed a bucket to catch bags of coins dropped by a helicopter. The helicopter was occluded by clouds, but the bags were directly observable to the participant and bag locations on each trial were drawn from a normal distribution centered on the helicopter with a standard deviation of 30 screen units (the entire screen width was 800 screen units). Since the helicopter was not directly observable, participants were required to infer its location based on previous bag locations (Figure 1a). For each block of 120 trials, the helicopter could only take two possible positions, one on the left side of the screen and one on the right side of the screen. For most trials, the helicopter would stay in the same location, but occasionally (1 in 8 chance after 5 “safe” trials) it would shift to an alternate location on the other side of the screen (Figure 1b). Thus, participants were required to infer the position of the helicopter amid occasional alternations in its “latent state”. Note that here we use latent state to mean the true underlying state of the world: which of two helicopters, differing in their location, is currently dropping bags. A major question of this research is whether some or all individuals form internal latent state representations that match this underlying structure, enabling them to rapidly react to transitions in the active helicopter location. In order to aid in formation of appropriate structural representations participants were informed before completing the task that there were exactly 2 helicopters. Each participant performed 2 blocks of 120 trials of the predictive inference task.

### 2.2 Behavioral analysis

The bucket placement on each trial reflects the participant's prediction about where the bag will land, and thus prediction error δ can be computed as the difference between the bag location and bucket placement for each trial. Similarly, a single trial measure of prediction updating (update) was computed as the difference between bucket placements on consecutive trials. Intuitively, the relationship between updating and prediction errors provides a measure of how much participants adjusted their predictions in response to new information. If the updates exactly match the prediction errors, then the participant completely adjusted their predictions to match the most recent bag position. In contrast, if updates are unrelated to prediction errors, then the participant was not influenced by the most recent bag position at all. In general, the slope of the relationship between updates and prediction errors captures the degree to which participants relied on the most recent bag position when providing an updated prediction, and we refer to this slope as the learning rate (Nassar et al., 2010). We examined the learning rate and update behavior using both descriptive models (learning rate model, population weight model, and individual weight model) as well as computational models of behavioral strategies (Rescorla-Wagner, ideal observer, and change point).

### 2.3 Learning rate descriptive model

To compare learning rates as a function of participant and model errors, we fit generalized-gaussian mixtures of two linear prediction error relationships to the update data (*U*) from each participant/model simulation separately according to the following equations:


$$\begin{array}{l} U=\alpha_{u}\delta *W+\alpha_{s}\delta *\left(1-W\right)\\ W=e^{-{\left(\frac{\delta }{300}\right)}^{10}} \end{array}$$

where δ is the prediction error (distance of the bucket from the prior coin), α_*u*_ is the learning rate (slope) for large errors, and α_*s*_ is the learning rate (slope) for small errors. The learning rate as a function of error is the first derivative of *U* with respect to δ. The generalized-gaussian weighting function *W* was chosen to have a relatively flat region spanning updates in positions that were likely to be within a single state. The model was fit using the MATLAB function *fmincon*. By fitting this model, errors can be smoothly interpolated for each participant aiding in comparison across participants/models. The difference between α_*s*_ and α_*u*_ was computed for each participant/model simulation group and compared using a Wilcoxon rank sum test.

### 2.4 Population weight descriptive model

A key advantage of our behavioral task is that participants could, in theory, learn the position of the alternate helicopter location and go to that position immediately after a transition, rather than learning incrementally from new bag locations. In order to examine this, we developed an analysis that focused on the second trial following a transition. For this trial, the influence of the true helicopter and bag positions on updates was estimated using data from all participants/model simulations according to the following equation:


$$\begin{array}{l} T_{i}=W_{h,i}H_{i}+W_{c,i}C_{i} \end{array}$$

where *i* indicates how many times the state has been seen previously, *T*_*i*_ is the update on the second trial after a state transition, *H*_*i*_ is the position of helicopter in the state, *C*_*i*_ was the bag position on the first trial following the transition, *W*_*h, i*_ is the weight of the helicopter position on the update, and *W*_*c, i*_ is the weight of the bag position on the update. The weight model combined data across participants, but was fit separately for each return *i* and the weights restricted to be between 0 and 1 using the MATLAB function *lsqlin*. Thus, our fitting procedure allows us to examine how *W*_*h*_ and *W*_*c*_, or the influences of the observed bag and remembered helicopter location, change with additional latent state repetitions. In particular, we surmised that participants would not be able to capitalize on knowledge of the helicopter locations at the beginning of the task block, but with repeated experience of those locations, would become better able to capitalize on that knowledge. Confidence intervals were computed by bootstrapping with 2000 samples. Variation in weights with respect to *i* indicates the extent to which participants/models overall were able to learn the latent positions of the two helicopters.

### 2.5 Individual weight descriptive model

For the second trial following a transition, the influence of the true helicopter and bag positions on updates was estimated using data from each participant/model simulation separately according to the following equation:


$$\begin{array}{l} T_{i}=H_{i}+\left(C_{i}-H_{i}\right)\frac{\exp\left(-\tau i\right)+W_{c,o}}{1+\tau } \end{array}$$

where *i* indicates how many times the state has been seen previously, *T*_*i*_ is the update on the second trial after a state transition, *H*_*i*_ is the position of helicopter in the state, *C*_*i*_ was the bag position on the first trial following the transition, *W*_*c, o*_ is the initial weight of the bag position on the update, and τ captures the rate of change of the bag position weight across state returns. This model can accurately track how the bag weight will diminish across repeats and vice versa for how the helicopter weight should increase across repeats. The weight model was fit, with *W*_*c, o*_ and τ as free parameters, using the MATLAB function *fmincon* with the parameters restricted to positive values. Learning of the helicopter position was compared between participants/model simulations by computing the predicted helicopter weight after five transitions according to the following equation:


$$W_{h,5}=1-\frac{\exp\left(-5\tau \right)+W_{c,o}}{1+\tau }$$

The values of *W*_*h*, 5_ for each participant/model simulation group was compared using a Wilcoxon rank sum test.

### 2.6 Computational process models of learning

To better understand the mechanisms through which participants performed the predictive inference task, we considered a set of computational models that differed in their latent state assumptions. First, we fit a Rescorla-Wagner model (Rescorla and Wagner, 1972) that assumed all learning was ascribed to a single latent state. Then, we considered a set of more complex models stemming from the Bayesian ideal observer for the task. The first was a Bayesian ideal observer model that attributed learning to two distinct latent states, allowing it to rapidly re-calibrate after helicopter transitions and make reuse of knowledge about the alternate helicopter location. The second model recognized latent state transitions, but assumed that transitions were change points and thus never reused information, always relearning from scratch after each inferred helicopter transition. We then considered a model that parametrically varied between these two Bayesian models, and also considered a mixture model in which behavior was modeled as a mixture of Rescorla-Wagner updating and our parametric Bayesian model. We describe each model and our fitting processes in more detail below.

#### 2.6.1 Rescorla-Wagner (RW)

The Rescorla-Wagner rule, also sometimes referred to as the delta rule, incrementally updates beliefs about the helicopter position according to prediction errors (Rescorla and Wagner, 1972):


$$B_{t+1}=B_{t}+\alpha \delta$$

$$\delta =x\left(t\right)-B_{t}$$

where *B* is the belief about the helicopter position on each trial, *x* is the bag position on each trial, δ is the observed prediction error, and α is the learning rate. Here we consider the simplest form of RW, where the learning rate (α) is assumed to be constant. This formulation is consistent with the idea that all information is treated equally and used to update a single representation of helicopter location, in contrast to the other models described below. It is worthy of mention that our Rescorla-Wagner model fit α as a single fixed value and did not incorporate any of the task dynamics described above in our descriptive behavioral analysis of participant learning.

#### 2.6.2 Two latent state ideal observer (IO)

The ideal observer model for our task determines the most probable location of the helicopters based on the entire history of bag positions using Bayesian inference over two latent states (Yu et al., 2021). To do so, the model considers all possible sequences of latent states (i.e. which helicopter is dropping the bags) that could have occurred up until the current trial. Conditional on the preceding state sequence, the model uses Bayes' rule to update a posterior probability distribution over positions for the helicopter *P* corresponding to the active state on each trial which we denote as follows:


$$\begin{array}{l} p\left(\mu |S_{i},x_{1:t+1}\right)=\frac{p\left(x_{t+1}|\mu \right)p\left(\mu |S_{i},x_{1:t}\right)}{p\left(x_{t+1}\right)}\\ =\frac{N\left(x_{t+1}|\mu ,\sigma \right)p\left(\mu |S_{i},x_{1:t}\right)}{p\left(x_{t+1}\right)} \end{array}$$

where μ reflects the helicopter location, σ reflects the standard deviation of the bag distribution (30 in our task/model), *S*_*i*_ reflects a specific sequence of states and *x*_*t*+1_ reflects the most recently observed bag location. Thus, in the model, each state sequence will do Bayesian belief updating over the two helicopter positions, however, they will differ in the exact trials that are used to infer the position of helicopter 1 as opposed to helicopter 2, as each sequence defines a different mapping between bags observed and the helicopter that they came from. This leads each sequence to have a different inference about where the two helicopters are located, and thus where upcoming coin bags should be expected to land. The latent state ideal observer model leverages these differences in order to compute a posterior probability distribution over possible state sequences and to update it on each trial according to the likelihood with which each assumed sequence would have given rise to the newest bag location. The likelihood of a given state sequence *S*_*i*_ producing an outcome μ is:


$$\begin{array}{l} l=p\left(x_{t+1}|S_{i},x_{1:t}\right)=\underset{\mu }{\sum }p\left(x_{t+1},\mu |S_{i},x_{1:t}\right)\\ =\underset{\mu }{\sum }p\left(x_{t+1}|\mu \right)p\left(\mu |S_{i},x_{1:t}\right)=\underset{\mu }{\sum }N\left(x_{t+1}|\mu ,\sigma \right)p\left(\mu |S_{i},x_{1:t}\right) \end{array}$$

Thus, the likelihood of a given state sequence *S*_*i*_ is defined by its posterior probability distribution over the active helicopter position *p*(μ|*S*_*i*_, *x*_1:*t*_) and the degree to which that posterior probability distribution aligns with the observed outcome $N\left(x_{t+1}|\mu ,\sigma \right)$.

In order to model possible state transitions, at the end of each trial, each possible state sequence gives rise to two “children”, one for each possible new state on the current trial (helicopter 1 now active or helicopter 2 now active). These updated state sequences inherit the probability of their “parent node” according to the task transition function:


$$\text{Same state:}L_{new}=Ll\left(1-h\right)$$

$$\text{Different state:}L_{new}=Llh$$

where *h* is a task hyperparameter describing the probability of a state transition (hazard rate) which was set to 0.125. As the number of possible state sequences scales exponentially with the number of observed bag drops (2^*t*^), and since the performance of model behavior was well approximated by considering a smaller number of sequences, we pruned the list of state sequences to consider only the 10 most probable ones at the end of each trial.

The key distinction between the IO model and the RW model is that the IO model can recognize latent state transitions and respond to them rapidly, by reusing previous knowledge about the location of the newly activated helicopter.

#### 2.6.3 Change point (CP) IO

The CP model is identical to the IO model except that the posterior distribution over helicopter location for the “inactive” helicopter is reset to uniform on each trial, meaning that a switch to the other state in this model is analogous to relearning from scratch (Wilson et al., 2010).

#### 2.6.4 Forgetful IO

The Forgetful IO model is an intermediate between the latent state and change point ideal observer models. Specifically, instead of resetting the beliefs of the inactive helicopter position completely, the forgetful model assumes that uncertainty is added to the representation of the inactive helicopter position (*P*) by the following equation:


$$P_{new}=P*\frac{1}{F\sqrt{2\pi }}\exp\left(-\frac{1}{2}{\left(\frac{x}{F}\right)}^{2}\right)$$

where *F* is the forgetfulness parameter, or more formally the standard deviation of Gaussian mean-zero drift that we assume gets added to beliefs about the inactive helicopter position on each timestep. Thus, *F* controls the degree to which the model resembles change point or latent states behavior with smaller values indicating a more latent states-like model.

#### 2.6.5 RW-IO combinations

One other possibility is that participants compare predictions from RW and IO style processes to make decisions on each trial. The RW component provides information on recent evidence while the IO component allows for integration across many trials to learn the mean position in one or both states. Modeling either process in isolation may not completely account for behavior on the task.

The RW-IO combination models assume that participants weight the output of the RW and IO style processes in determining the probability of selecting each option. The parameter *W*_*IO*_ (mixing weight) indicates the degree to which the IO style model's output determines the bucket position:


$$B_{t+1}=W_{IO}B_{t}^{IO}+\left(1-W_{IO}\right)B_{t}^{RW}$$

Note that this model, which we refer to throughout the text as “forgetful-IO”, is the most complex and can account for data generated from any of the simpler models under different parameter settings. In particular, learning onto a single latent state, which was achieved by the RW model, is afforded when the mixing weight of the forgetful-IO model is set to zero. Learning onto two latent states is achieved when the mixing weight is set to one and the forgetfulness parameter is set to zero, such that the ideal observer portion of the model retains all information about the location of a given helicopter over the period in which that helicopter is not active. Finally, learning onto greater than two states, consistent with treating each transition as a changepoint, is achieved when the mixing weight is set to one and the forgetfulness parameter is set to a high value, effectively erasing memory about the location of a given helicopter during the period over which it is inactive.

We tested combination models for all three IO variants for a total of seven different computational models.

### 2.7 Model fitting

We fit each model by minimizing sum of squared error (SSE) between the model predictions and participant behavior using the MATLAB function *fmincon* with 50 random initialization points to avoid local minima. Model parameters were scaled to a similar range. To account for autocorrelated errors in the fit, a state-dependent AR(1) model was fit and removed from the error time series before computing the SSE. Models were fit separately for each of the two runs of the task with the final SSE corresponding to the out-of-sample error using the best fitting parameters from the unused run. Best fitting model parameters and autoregressive parameters were reported for a fit using both runs. The noise in the model was computed using the standard deviation of the residuals ignoring extreme outliers.

### 2.8 Model selection

The optimal model was chosen based on Bayesian model selection (Stephan et al., 2009) with log model evidence *p*(*y*|*m*) estimated using the following equation:


$$\begin{array}{l} p\left(y|m\right)\approx -\frac{n}{2}\log\left(\frac{SSE_{m}}{n}\right) \end{array}$$

where n is the total number of trials and *SSE*_*m*_ the out-of-sample sum of squared error for model *m*. Models were evaluated based on the expected probability the data from a random participant was generated by the model *r*_*m*_. We decided to go forward with the Forgetful-RW model as it had the greatest *r*_*m*_ values.

Code for all models and model fitting is freely available on Github: https://github.com/learning-memory-and-decision-lab. All data will be made available upon request.

### 2.9 Model validation

The Forgetful RW model was validated in three ways: test-retest reliability of model parameters and strategy assignment (RW, CP, IO) between the two runs of the experimental design, parameter recovery using the best fitting parameters, and posterior predictive checks with the same behavioral metrics discussed above but split over the three strategies.

### 2.10 MRI acquisition

Before completing the task, resting-state fMRI scans were acquired from a total of 265 right-handed participants recruited from Southwest University in China. During the scanning session, participants were instructed to keep their mind relaxed, eyes open, and avoid any movement. MRI procedures have been previously described elsewhere (Zhang et al., 2022). However, in brief, MRI data were acquired using a Siemens 3T scanner (Siements Magnetom Trio TIM, Erlagen, Germany). An MPRAGE sequence was used to collect structural images (128 slices; repetition time (TR) 2,530 ms; echo time (TE), 3.39 ms; flip angle, 7 degrees). Resting state functional images were acquired with a T2 weighted echo planar imaging (EPI) sequence (resolution sequence = 64 × 64; voxel size 3.1 x 3.1 x 3.6; TR = 2,000 ms; TE = 30 ms; flip angle = 90 degrees). Resting state scan lasted 12 minutes and collected 360 volumes.

### 2.11 MRI preprocessing

Anatomical scans were segmented into gray matter (GM), white matter (WM) and cerebrospinal fluid (CSF), non-linearly aligned to MNI space using the DARTEL algorithm. Resting state data was preprocessed as follows: (1) first ten volumes were discarded, (2) remaining 350 volumes were slice timing and motion corrected, (3) co-registered with anatomical scans and segmented into GM, WM and CSF, (4) normalized to MNI space with 3 × 3 × 3 mm cubed voxels, (5) spatially smoothed with a Gaussian Kernal (4mm FWHM), (6) WM, CSF, global signal, and 24 motion parameters were regressed out of each voxel timeseries, (7) band pass filtered (0.01-0.08 hz) and linear detrended, (8) volumes with framewise displacement greater than 0.2mm were removed, along with the preceding and subsequent volumes.

### 2.12 Resting state fMRI data

After pre-processing we compute functional connectivity between brain nodes to be used as input variables for the analysis. Specifically, the behavioral outcome was a binary variable with 131 participants in the RW group and 134 in the non-RW group, representing individuals who tend to adjust beliefs based on recent evidence and utilize deeper causal structures, respectively. Each participant had a time series of gray-scale 2D scans, from which BOLD time series were obtained for each anatomical seed region. The analysis was conducted on a total of 264 regions of interest (ROIs), which were defined based on the (Power et al., 2011) parcellation, with each ROI being modeled as a 10 mm diameter sphere. The voxel-level time courses in each ROI were spatially averaged to obtain 264 time courses for each participant.

Let *Y*_*ikt*_ be the BOLD signal for participant *i* of brain region *k* at time *t*, where *i* = 1, ..., *N*, *k* = 1, ..., *V*, and *t* = 1, …, *T*_*i*_. In this application, the sample size is *N* = 265 and the total number of ROI is *V* = 264. The total time length of the scan, *T*_*i*_, varied among participants, but was mostly around 300 time points. Therefore, for each participant, we had a matrix of dimension *T*_*i*_×*V*, with each column representing the BOLD time course of a brain region. We calculated the pairwise Pearson's correlation coefficient of BOLD signal time courses between all pairs of regions *k, k*′:


$$r_{k,k^{\prime }}=cor\left(Y_{ik•},Y_{ik^{\prime }•}\right),$$

resulting in a 264 × 264 correlation matrix representing the functional connectivity matrix. We applied Fisher's Z transformation to the raw correlation coefficient $r_{k,k^{\prime }}$, which is defined by Fisher (1915) as follows.


$$\begin{array}{l} z=\frac{1}{2}\log(\frac{1+r_{k,k^{\prime }}}{1-r_{k,k^{\prime }}}). \end{array}$$

Finally, we aggregated the correlation data using the Power et al. (2011) network assignments. Specifically, for the default mode, cingulo-opercular, ventral attention, visual, fronto-parietal, salience, and dorsal attention networks, we computed average correlations within the network and average correlations with other networks. This produced a total of 28 (i.e., 1/2 × 7 × (7+1)) resting state features, which we subsequently used in regression models attempting to predict participant behavior.

### 2.13 Behavior classification from resting state data

As discussed further in the results section, we fit standard logistic regression models using principal components (PCs) of our raw correlation data, the correlation data aggregated by network, and aggregated network PCs as predictors. The PCs themselves were computed outside the CV folds in order to minimize overfitting (this does introduce some minor non-independence of folds, which would only be a potential concern if we reported marginal positive results rather than nulls). To assess predictive performance, we also performed regularized logistic regression analyses using cross-validation. All models were fit after standardizing (i.e., z-scoring) predictor columns reflecting resting state features, which was done by fitting the z-score transform on the training set, applying to the relevant fold, then applying at prediction time to the held out data. Logistic regression models were fit using the Logit class from the statsmodels package in python, whereas the regularized models were fit using sklearn's LogisticRegression class.

In each fit (including the leave-one-out cross-validation folds) we balanced class distributions via bootstrap resampling. So, for example, if data-to-be fit had 10 positive cases and 7 negative cases, we would sample our data set (with replacement) for 3 negative cases and fit the model on the resulting total of 20 cases. As the class distributions were highly imbalanced (73 IOLike cases, 133 RWLike cases, 59 CPLike cases) this resulted in substantial resamples of minority classes (we also verified that our results did not meaningfully change on the basis of loss reweighting instead of class resampling).

The PCs (and therefore, the resulting subject scores) were computed using a consensus-based bootstrapping approach. Specifically, we resampled (with replacement) 100 synthetic data sets of 265 participants, and for each of these datasets we computed principal components using sklearn's PCA class. We aligned the principal components using a greedy matching algorithm based on the correlation matrix relating the dataset's PCs to the original (non-synthetic) data. Specifically, we associated synthetic dataset PCs to original PCs by iteratively matching the top-most correlated vectors. During this process, we also computed participant scores (on the PCs) and assembled a list of these for each participant. If a participant appeared more than once in a dataset, we only appended one copy of their scores. As a result, we were able to obtain bootstrapped consensus PCs (via averaging), correlations between PC resamples, signal to noise ratios for loadings, and signal-to-noise ratios for subject scores. The score and loading SNRs were computed as absolute values of means divided by standard deviations.

## 3 Results

### 3.1 Behavioral results

**Figure 2:**
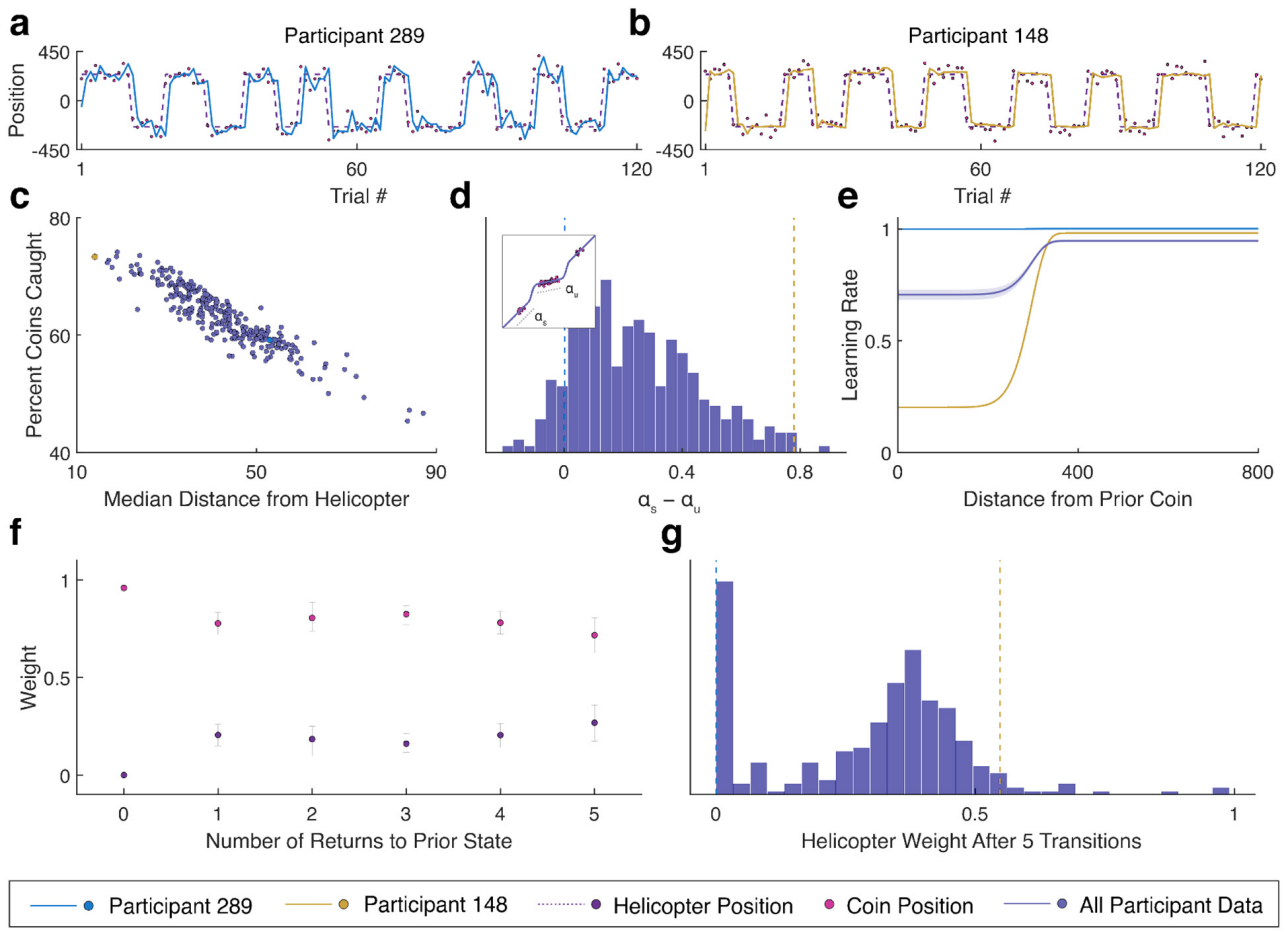
Participants demonstrate a range of behaviors across several metrics. **(a, b)** Example task behavior from two participants with coin positions indicated by the pink points, helicopter position by the dashed purple line, and bucket positions from participant 289 **(a)** in light blue and 148 **(b)** in yellow. Data corresponding to the two participants are shown in blue and yellow respectively in subsequent plots. **(c)** Scatter plot of the percent of bags caught against the median distance from the active helicopter across all trials for each participant. **(d)** Histogram of the difference between the learning rate within a state (*a*_u_) and during state transitions (*a*_s_) (illustrated in the inset) for each participant. **(e)** Learning rate vs. the distance from the prior bag averaged across participants (purple) with the 95% confidence interval indicated by the lighter outline. **(f)** Scatter plot of the weight of bag (pink) and helicopter (purple) position on subsequent trials following a state change using data from all participants. Bootstrapped 95% confidence intervals are indicated by the gray error bars. **(g)** Histogram of the helicopter weight after five transitions predicted using a model fit to each participant separately.

Participants demonstrated a range of behaviors when inferring the location of the helicopter. Some participants followed the locations of the falling bags directly and frequently updated the bucket position (Figure 2a). However, others inferred stable locations for the helicopter and only updated the bucket position following large changes in the locations of the falling bags (Figure 2b). These strategic differences had direct effects on performance with participants that better inferred the position of the helicopter receiving more rewards than those that simply followed the bag locations (Figure 2c). We sought to quantify behavioral correlates of these strategies by assessing differences in belief updating—the adjustments in bucket position from one trial to the next. We analyzed updates by fitting regression models to each participant's prediction errors that accounted for differences in learning rates for small and large errors that might be expected when a helicopter was stable or changing states (Figure 2d inset). Model fits showed a wide range of differences in belief update behavior between stable and changing states (Figure 2d) with the group average suggesting proportionally smaller updates when the helicopter was stable (Figure 2e). We additionally analyzed updates at state changes with linear regression models that assessed how much the bag and true helicopter positions influenced the update. Initial updates were entirely made based on the bag locations with subsequent returns to a state becoming increasingly informed by the true position (Figure 2f). Participant weighting varied heavily with some participants never making updates consistent with the true helicopter position whereas others showed clear evidence of inference (Figure 2g).

### 3.2 Computational modeling

**Figure 3:**
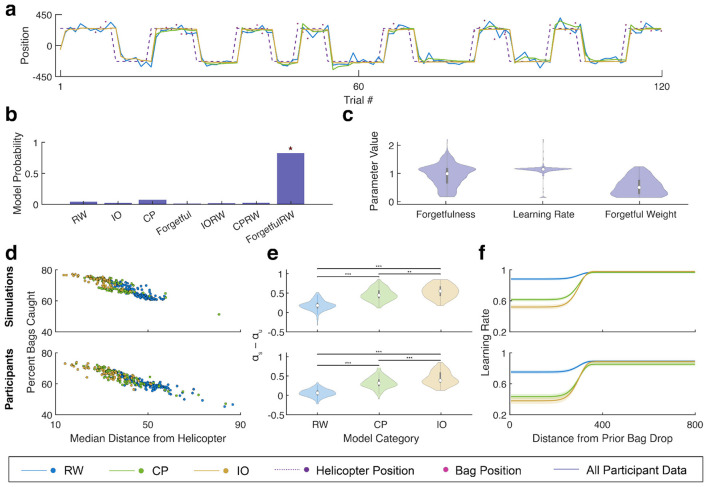
The range of participant behaviors are well described by a combined Forgetful Ideal Observer/Rescorla-Wagner model. **(a)** Example task behavior from RW (blue), CP (green), and IO (yellow) models with bag positions indicated by the pink points and helicopter position by the dashed purple line. **(b)** Bar chart of the expected probability that each model generated the data for a randomly selected participant (r). Forgetful-RW had the greatest exceedance probability which is indicated by the red star. **(c)** Violin plots showing the range of values fit to participant data for the three parameters of the Forgetful-RW model. The median for each parameter is indicated by the white point with the thick gray bar showing the interquartile range. **(d–f)** Participants were divided into three groups based on values of the forgetfulness and forgetful weight parameters with groups for more RW (blue), CP (green), and IO (yellow) like behavior. Data simulated for the best fitting parameters for each participant are shown in the top row and data from each participant are shown in the bottom row, both split based on the groupings. **(d)** Scatter plot of the percent of bags caught against the median distance from the active helicopter across all trials for the three grouping of simulations and participants. **(e)** Violin plots of the difference between the learning rate within a state and during state transitions for the three grouping of simulations and participants. Significant differences between groups are indicated by stars (Wilcoxon Rank-Sum test, *p < 0.05, ***p < 0.0005). **(f)** Learning rate versus the distance from the prior coin averaged across simulation and participant groupings with the 95% confidence interval indicated by the lighter outline.

To better dissect differences in participant strategies, we fit a series of computational models to participant choice data. We considered four types of models: a Rescorla-Wagner (RW) learning rule where updates in bucket position were directly proportional to prediction errors, an ideal observer (IO) model that performed Bayesian inference to assign observations to one of two learned states, and a change point (CP) model which used Bayesian inference to predict the mean position from the noisy bag locations and reset after each transition (Figure 3a). To fully describe the range of participant behaviors, we additionally considered a hybrid “forgetful” model, that categorized experience into two latent states, but slowly “forgot” the associations between inactive latent states and outcomes, as well as mixed RW-Bayesian models.

Of the 7 models we fit, the mixed Forgetful-RW model had the greatest exceedance probability across the entire group (Figure 3b, Supplementary Table S1). Given that the mixed Forgetful-RW model could produce behavior consistent with any model in our set (see methods) and that it provided the best fit to participants, we performed all further analyses using it. Forgetful-RW parameters were consistent with this decision showing a wide range of mixing weights and forgetfulness–the two parameters controlling the overall model behavior (Figure 3c). To compare simulated model data to participant data, we split participants into three groups based on the mixing weight and forgetfulness. The RW group consisted of all participants with mixing weights below the median while the remaining participants were split into CP and IO groups based on their forgetfulness parameters relative to the median respectively (2:1:1 group ratio). This split was made based on the observation that most participants showed predominantly RW-like behaviors (Figures 2d, g). IO weight and strategy groups were consistent between the two-runs and when refit via parameter recovery. Learning rate and forgetfulness had comparably lower consistency, but this was unsurprising considering their impact on model behavior would depend on the magnitude of the IO weight (Supplementary Figures S1, S2). Participants and models in the IO group received the most rewards and remained closest to the true helicopter position, followed by the CP group, then the RW group (Figure 3d). IO participants and models had significantly greater (Wilcoxon Rank-Sum test, *p* < 0.05 CP, *p* < 0.0005 RW) differences between learning rates during stable periods and state changes compared to the RW and CP groups (Figures 3e, f). The CP group also had significantly greater differences compared to the RW group (Wilcoxon Rank-Sum test, *p* < 0.0005) (Figures 3e, f). No significant differences were observed between the model groupings for reaction times on each trial (Supplementary Figure S3).

**Figure 4:**
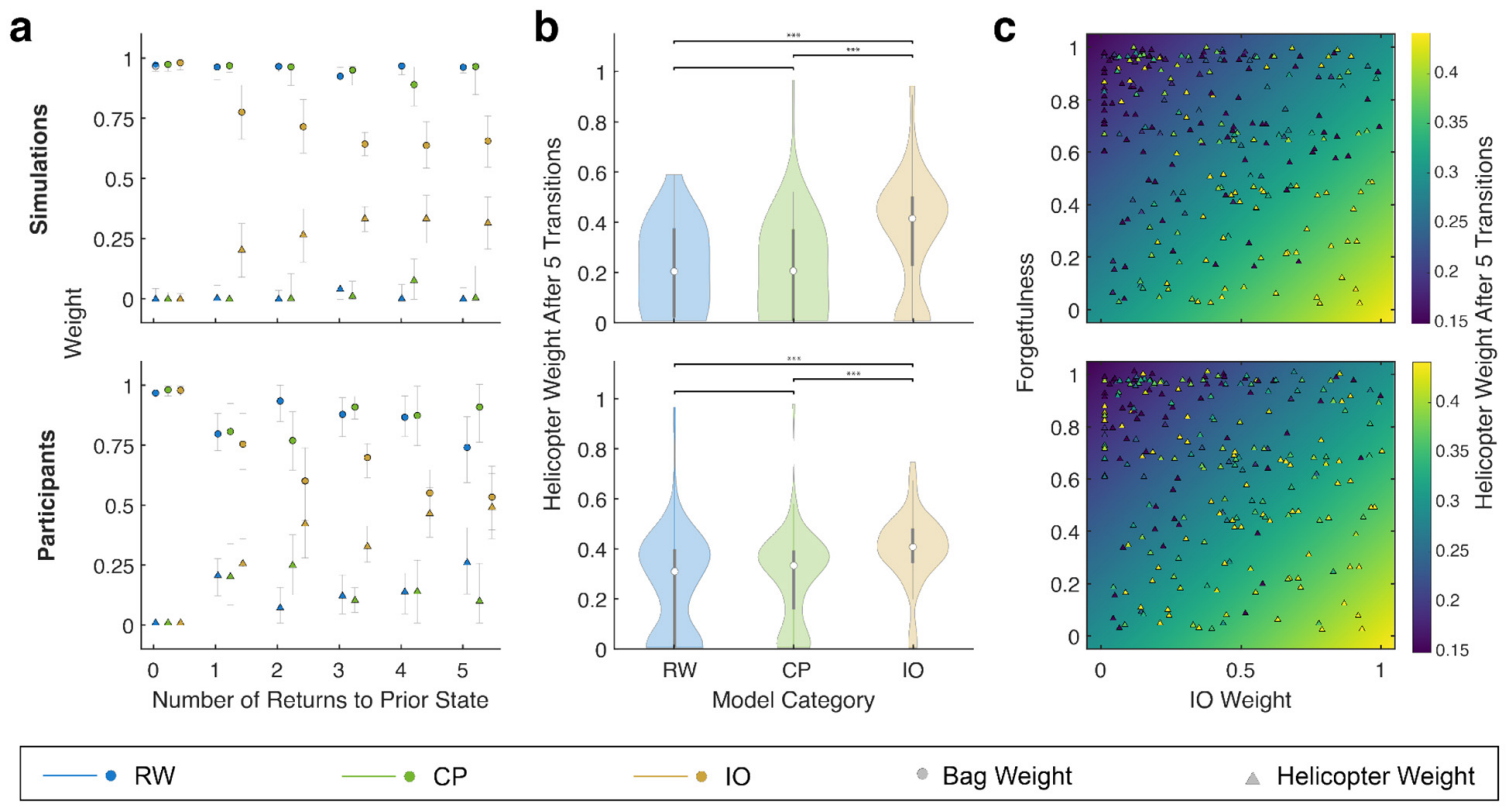
Participants best fit by a more IO-like model demonstrate features of latent state learning. **(a–c)** Participants were divided into three groups based on values of the forgetfulness and forgetful weight parameters with groups for more RW (blue), CP (green), and IO (yellow) like behavior. Data simulated for the best fitting parameters for each participant are shown in the top row and data from each participant are shown in the bottom row, both split based on the groupings. **(a)** Scatter plot of the weight of bag (circle) and helicopter (triangle) position on subsequent trials following a state change using data from all simulations or participants in each group. Bootstrapped 95% confidence intervals are indicated by the gray error bars. **(b)** Violin plots of the helicopter weight after five transitions predicted using a model fit to each participant or simulation separately divided into the three groups. Significant differences between groups are indicated by stars (Wilcoxon Rank-Sum test, ***p < 0.0005). **(c)** Scatter plot of the helicopter weight after five transitions as a function of the IO weight and forgetfulness for simulations and participants. More yellow points correspond to larger helicopter weights while more blue points correspond to smaller helicopter weights. A linear model is fit to the data and shown beneath the points.

Next, we sought to assess whether there were individual differences in latent-state learning that were supported by our computational model. Using the same RW, CP, and IO groups, we assessed the weighting of bag location and the true helicopter position on updates at state transitions. Participants and models in the IO group showed increasing weighting of the true helicopter position with subsequent returns to the corresponding state. In contrast, the CP and RW groups showed no evidence of weighting the helicopter position when updating position at state changes (Figure 4a). The final weight of the helicopter position on the update after 5 repeated returns to each state was significantly greater for the IO group compared to the RW and CP groups for both participants and models (Wilcoxon-Rank Sum test, *p* < 0.0005) (Figure 4b). Additionally, there was no significant difference between the CP and RW groups (Wilcoxon-Rank Sum test, *p* > 0.05) (Figure 4a). This result suggests that only a subset of the participants actually successfully identified the true latent states in the environment and could be identified based on their best-fitting model parameters. Lastly, we fit a linear regression model to the final helicopter weight as a function of the forgetfulness and mixing weight parameters. Regression fits showed that participants and models had increasingly greater weighting of the helicopter with increasing mixing weight and decreasing forgetfulness (Figure 4c).

### 3.3 fMRI-based prediction results

We tested whether study participants' resting state functional connectivity could be used to predict their behavior, in the form of best fitting model classes. To make these predictions, we chose to train and test individual binary classifiers of IOLike, RWLike, and CPLike class membership, using logistic regression. This separate prediction approach has the practical advantage, over training 3-way classifiers of allowing for more straightforward analysis and interpretation. We took several different approaches to prediction, using either PCs of the resting-state correlations, correlations aggregated by functional network (such as default mode or cingulo-opercular networks; see the methods section) or PCs of the functional network aggregates as predictors. We describe the predictors in more detail below, and in the methods section.

**Figure 5:**
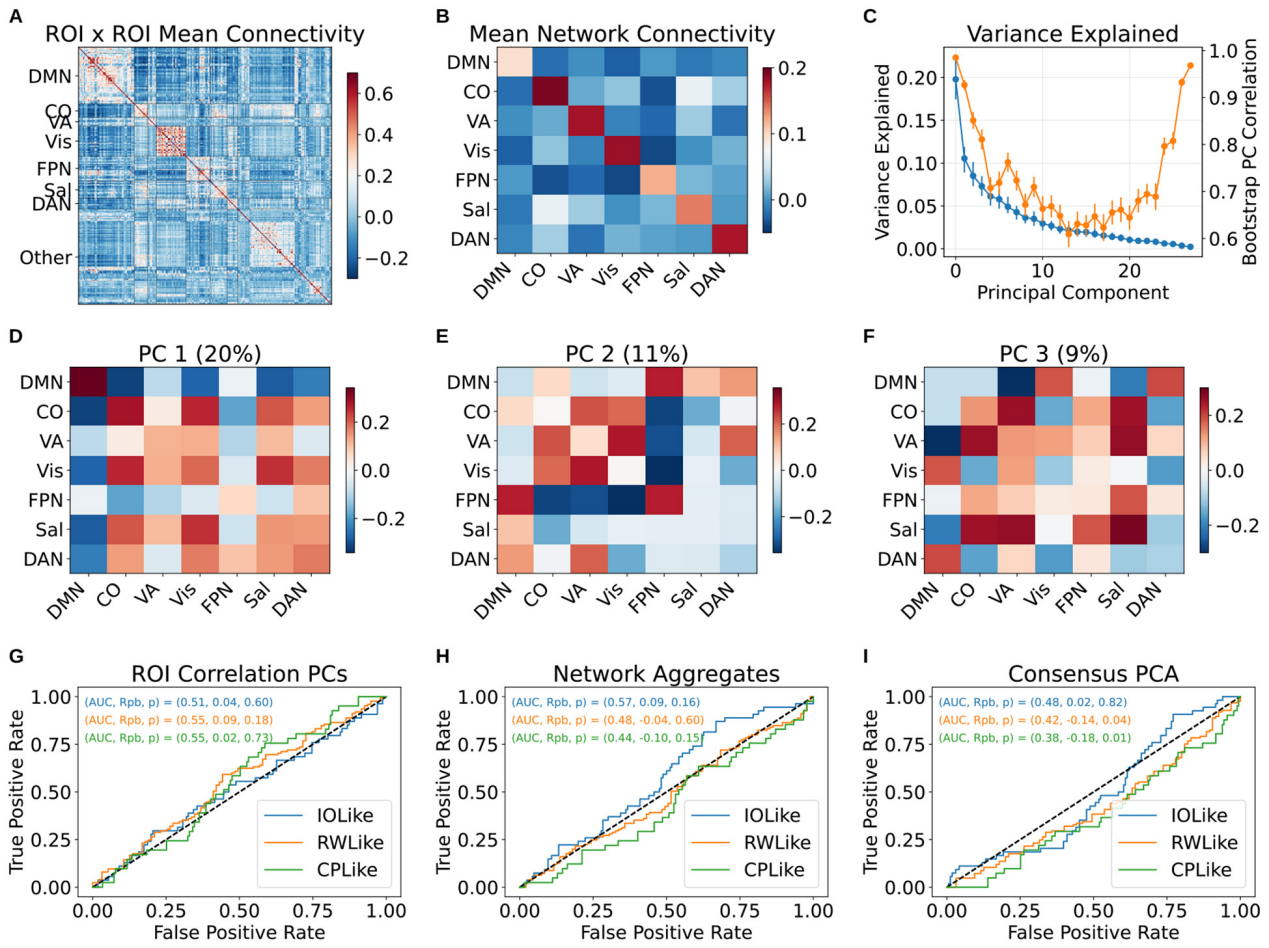
Lack of ability to predict subject computational classes from rs-fMRI data using logistic regression models of class membership. **(A)** Plot of raw ROI correlations, labeled by network. **(B)** Average within and between network connectivity (averaged correlation coefficients). **(C)** PC variance explained over per-subject instances of B, along with PC reliability. **(D–F)** First three PCs. PC1 primarily reflects DMN anti-correlation with other networks. **(G)** Receiver Operating Characteristic curve showing true positive rate as a function of false positive rate across class decision-boundary values for a regularized principal-component logistic-regression using the 77 top ROI correlation matrix PCs explaining just over 50% of the ROI correlation-matrix variance. None of the outcome variables are significantly predicted, as can be seen by observing the AUCs around 0.5 and the low point-biserial correlations. **(H)** Similar to A, using a network-aggregated correlation matrix representing functional connectivity within and between the networks from Power et al. (2011). In this case, we did not use principal components of the data, and merely fit elastic net regressions using the reduced correlation matrix entries. **(I)** Similar to B, using PCs of the functional-network data which we were sure we had estimated with reasonably high accuracy (loading SNR > 1.0 assessed over bootstrap resamples).

None of the regression models produced class predictions exceeding chance performance. In the first analysis (Figure 5G), we used the full correlation matrix across our 264 ROIs (264 × 265/2 independent entries; Figure 5A) to predict subject classes by first performing a PCA on the correlations, taking the top PCs explaining 50% of the variance (of which there were 77), and entering these into a leave-one-out cross-validated elastic-net logistic regression. The results for the three classes, specifically ROC curves for the held-out cases, are shown in Figure 5G. The AUCs for each class were roughly 0.5, and point-biserial correlations between predicted class membership probabilities and actual class memberships were approximately zero and were not statistically significant.

To determine whether we could obtain better models by removing noise from the data, we considered two more sophisticated modeling strategies. Both strategies relied on aggregating the data into functional networks using the assignments from (Power et al., 2011). Specifically, we computed within-network and between-network average correlations for each subject, by taking averages of blocks of the ROI correlation matrices (within-network averages would correspond with diagonal blocks in the network-grouped ROI correlation matrix, whereas between-network averages correspond with off-diagonal blocks). This reduced the data to a 28 x 28 correlation matrix representing functional connectivity within and between functional networks (Figure 5B). We repeated the elastic-net regression using the new predictors (results shown in Figure 5H) without observing any improvement in performance. As a final check, we ran a PCA on the network-level data, and used a subset of PCs as predictors (Figures 5C–F). We observed that the first network PC reflected isolation of default mode activity from the rest of the network, providing a basic sanity check (Figure 5D). Notably, most other PCs had somewhat limited reliability, however, as assessed using bootstrapped signal-to-noise ratios, subject-score distributions, and PC loading correlations over sample populations (correlations in Figure 5C). This suggested that the data was being de-noised reasonably effectively for some components, but may not have contained substantial information in most. When using these in the logistic regression model, we included only the PCs with a loading SNR > 1.0, of which there were 9, including the top 4 PCs. The resulting logistic regression did not improve over the others (Figure 5I) (the below-chance AUC in Figure 5I is a known, occasional consequence of high-variance estimation in cross-validated classification with weak or absent true signal; Jamalabadi et al., 2016).

To further probe the robustness of these null results, we conducted several additional analyses. First, following a reviewer suggestion, we excluded participants near the classification boundaries—specifically, those within 0.05 units of the median-split boundaries defining the behavioral classes—removing the most marginal 10% of participants in each classification group. This did not change the pattern of results, with AUCs near 0.5 across the raw PC, network-aggregate, and consensus PCA analyses. Second, we replaced the logistic regression with a continuous regression of the IO weight parameter on resting-state PCs, yielding point-biserial correlations near −0.1 and *p*-values near 0.2, consistent with noise. Third, we substituted random forest classifiers for logistic regression on both the network-aggregated data and the network-aggregated PCA-transformed data; AUCs remained near 0.5 with negligible correlations and evidently random *p*-values. Taken together, these additional analyses indicate that the null prediction result is not attributable to the choice of classification boundary, the use of categorical rather than continuous outcomes, or the linearity of the classifier.

## 4 Discussion

Reinforcement learning algorithms can learn very different things, depending on the way in which they represent states of the world. Here we examined behavior of a large cohort of individuals performing a learning task in which the true underlying state was not directly observable and alternated occasionally. We found individual differences in learning behaviors consistent with three different computational learning profiles. One of these profiles was best described by a Rescorla Wagner learning rule relying on a single fixed state. A second group was best described as combining learning with inference over two discrete states, whereas a third group also recognized state transitions, but did not reuse information from previous visitations to a state, and were best described by a latent state model that “forgot” about previously learned states. In terms of state representations, this could be thought of as a strategy in which individuals created a new state after each unexpected transition in the environment, allowing them to be sensitive to change, but preventing them from exploiting knowledge gained from previously visited contexts.

There are a number of ways to think about the three computational learning profiles that we observed. One way to think about the strategies is living on a continuum across model complexity (Gershman and Lai, 2021; Gershman, 2020; Fang and Sims, 2025; Filipowicz et al., 2020). One interpretation along these lines would be that individuals best fit with a single state model have the least complex world model, and that those with a two state have a slightly more complex one, and those who are resetting states at each transition point having the the most complex. Through this lens, individuals best-fit with the Rescorla-Wagner learning model might be viewed as using an overly simple world model, whereas those who relearn from scratch at each alternation could be viewed as an overly complex one. This tracks with common intuitions from machine learning regarding the bias variance tradeoff—with representations that are too simple leading to poor performance due to bias, and representations that are too complex leading to poor performance due to excessive variance (Glaze et al., 2018). The sweet spot is a representation just sufficiently complex to capture the features of the data, in this case that required two latent states.

However, there are also some problems, or at least untested assumptions in the interpretation above. First, those individuals who are best fit by a Rescorla-Wagner model tend to be updating fairly massively according to each new data point. The Rescorla-Wagner model fits best, but high learning rates suggest that these individuals are treating each new data point as a completely new situation—relying only on the most recent data and not integrating over the past. Thus, an alternative view of these participants might be that they have a state representation for which each time-point is its own unique state - providing a sort of maximally complex model that fails to generalize. Biologically inspired models of belief updating have highlighted the possibility that latent states could be encoded in persistently firing neural populations, with changes to the active population code effectively creating a new state and thus reducing the influence of prior learning on upcoming behavior (Razmi and Nassar, 2022; He et al., 2025). However, an alternative view of this behavior might be as a simple cognitive strategy - following the most recent outcome is a simple rule that is easy to implement and while not achieving perfect performance - may optimize something more resource rational, like performance per unit cognitive effort expended (Bruckner et al., 2025).

The idea that heterogeneous behavioral profiles reflect cognitive strategies, rather than relatively stable, biophysical constraints, matches well with our failure to identify connectivity patterns that reliably differentiate between groups of individuals. However, it is important to note some fairly major limitations on this front. First, it is not clear to what extent resting state connectivity measures should or do vary according to low-level biological constraints such as excitation/inhibition balance and NMDA receptor density that should, theoretically, affect the brain's ability to maintain a fixed context representation that could serve as a state for learning (Murray et al., 2017; Wang, 2001). Second, if resting state fMRI does provide a window into how individuals might stably differ, it is not clear that our data are sufficiently powered to observe it. Test retest reliability of resting state fMRI data is low (Marek et al., 2022) and our computational phenotyping is by nature probabilistic—raising questions as to whether our N of 265 is sufficient to observe meaningful differences in resting state connectivity related to our constructs of interest. Lastly, there are many other machine learning approaches and methods for assessing resting state functional connectivity that we did not specifically explore. It is possible that a deep neural network or an analysis of network properties and voxel based measures could yield different results. However, keeping these caveats in mind, our results certainly favor an interpretation of individual differences that is more transient and task specific, rather than one that is stable and linked to hard-wired features of brain function.

## 5 Conclusions

In summary, our results highlight considerable heterogeneity in belief updating strategies across individuals that could be characterized computationally in terms of the state representations that best describe them. We show that how people break their world into states affects the dynamics with which they learn about the world—and we failed to link these differences to reliable differences in resting state brain connectivity across individuals.

## Data Availability

The raw data supporting the conclusions of this article will be made available by the authors, without undue reservation.
