# Supplementary material for: Computational learning phenotypes are not related to individual differences in resting-state fMRI connectivity
Source: Front Neurosci. 2026 May 1;20:1720206. doi: 10.3389/fnins.2026.1720206 (PMC13176231; doi:10.3389/fnins.2026.1720206)
Supplement: Supplementary file 1 [file Data_Sheet_1.pdf]

Supplementary Data for: Computational learning phenotypes are not related to individual differences in resting-state fMRI connectivity

Evan Dastin-van Rijn[a], Linda Q. Yu[c], Dan Scott[c], Yifan Zhao[b], Ani Eloyan[b], Joseph W. Kable[d], Tingyong Feng[e], and Matthew Nassar[c,f]

[a]Department of Biomedical Engineering, University of Minnesota

[b]Department of Biostatistics, Brown University

[c]Department of Neuroscience, Brown University

[d]Department of Psychology, University of Pennsylvania

[e]Department of Psychology, Southwest University

[f]Robert J. and Nancy D. Carney Institute for Brain Science, Brown University

March 6, 2026

Model	Mean SSE	SE SSE	Behavior Probability	Exceedance Probability
RW	1.17×10^6	5.77×10^4	0.039	0
CP	1.19×10^6	5.11×10^4	0.07	0
IO	1.35×10^6	5.11×10^4	0.02	0
Forgetful	1.31×10^6	5.61×10^4	0.01	0
CP-RW	1.10×10^6	5.78×10^4	0.02	0
IO-RW	1.09×10^6	5.78×10^4	0.01	0
Forgetful-RW	9.87×10^5	5.22×10^4	0.82	1

Table S1: Model Fit Statistics

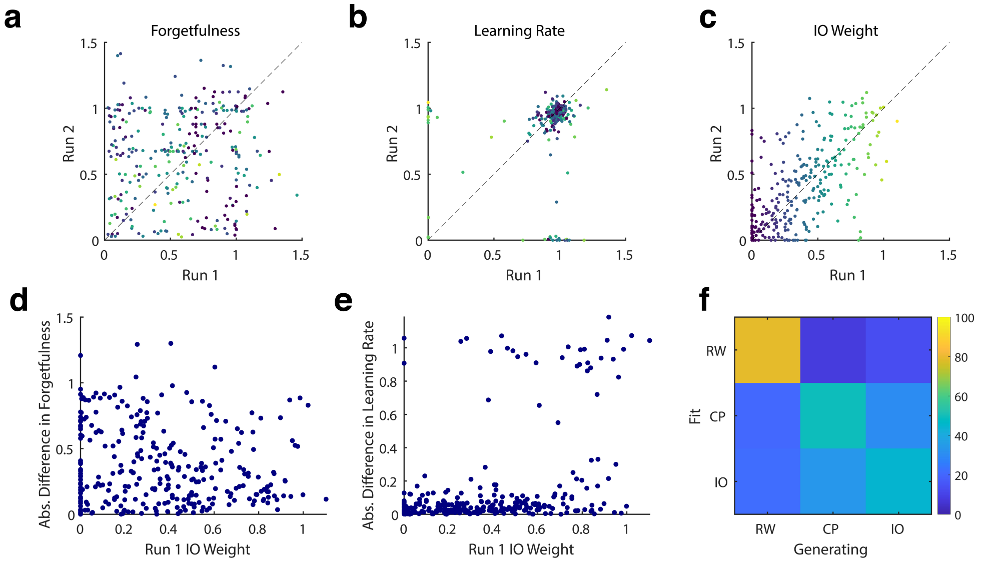

Figure S1: Test-retest reliability of Forgetful-RW model. (a-c) Correlations between run 1 and run 2 model parameters for (a) forgetfulness (R=0.24), (b) learning rate (R=0.25), and (c) IO weight (R=0.97). (d & e) Absolute differences in parameter values between the two runs were greater with low IO weight (d) for forgetfulness and high IO weight (e) for learning rate. (f) Strategy groupings were consistent between the two runs particularly when comparing RW strategies to latent state strategies (CP & IO).

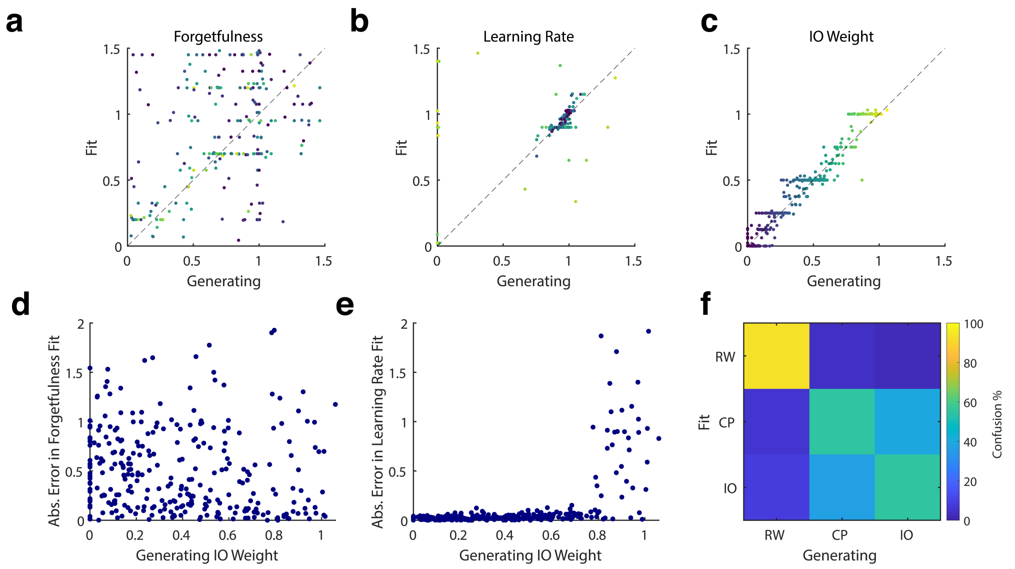

Figure S2: Parameter recovery of Forgetful-RW model. (a-c) Correlations between generating and fitting model parameters for (a) forgetfulness (R=0.10), (b) learning rate (R=0.29), and (c) IO weight (R=0.66). (d & e) Absolute errors in parameter values between the generating and fitting parameters were greater with low IO weight (d) for forgetfulness and high IO weight (e) for learning rate. (f) Strategy groupings were consistent between generating and fitting parameters particularly when comparing RW strategies to latent state strategies (CP & IO).

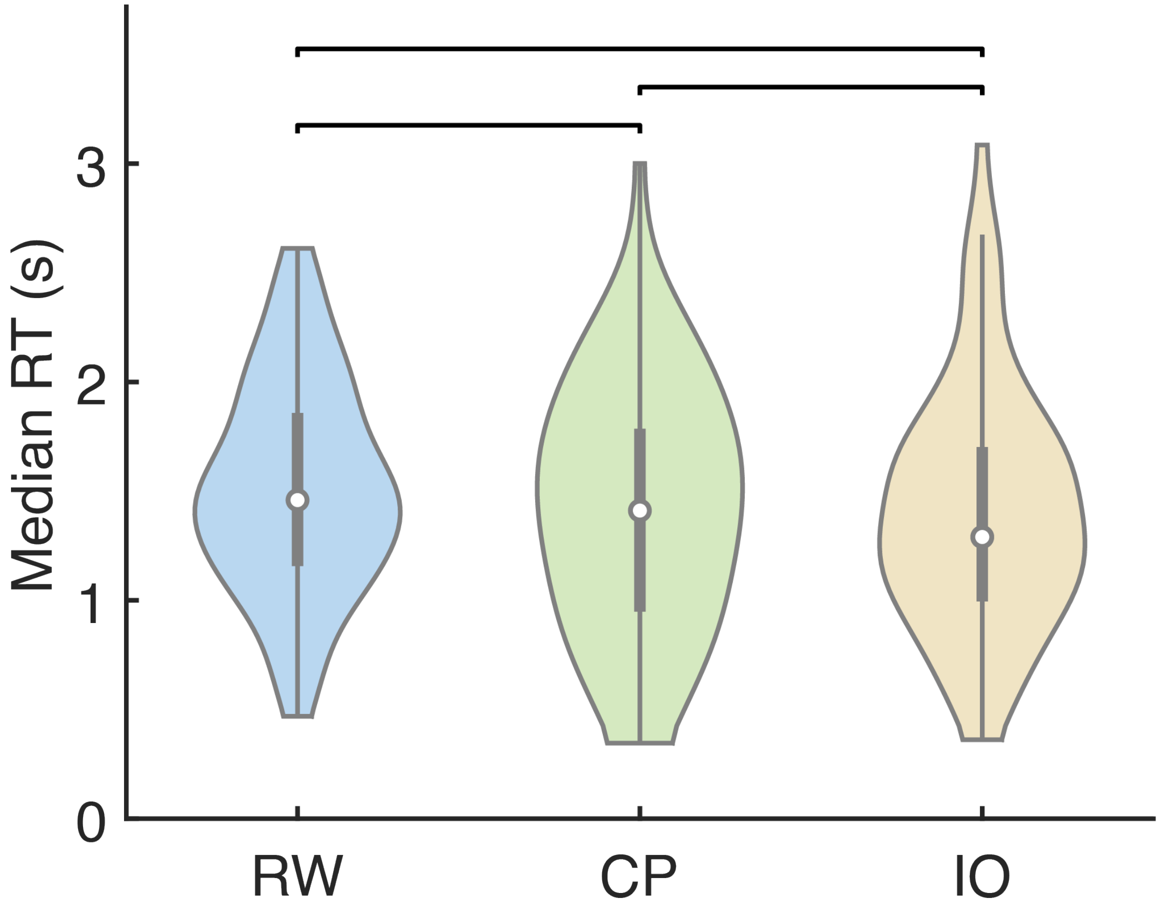

Figure S3: Model groupings had no relationship with reaction time. Participants were divided into three groups based on values of the forgetfulness and forgetful weight parameters with groups for more RW (blue), CP (green), and IO (yellow) like behavior. Violin plots are shown of the average reaction time for each participant split for the three grouping of simulations and participants. No significant differences between groups were observed.
